# Lower serum sodium levels predict poor clinical outcomes in patients with insomnia

**DOI:** 10.1186/s12882-020-02051-w

**Published:** 2020-09-05

**Authors:** Eunjin Bae, Tae Won Lee, Ha Nee Jang, Hyun Seop Cho, Sehyun Jung, Seunghye Lee, Se-Ho Chang, Dong Jun Park

**Affiliations:** 1 Department of Internal Medicine, College of Medicine, Gyeongsang National University and Gyeongsang National University Changwon Hospital , Changwon, South Korea; 2 Department of Internal Medicine, College of Medicine, Gyeongsang National University and Gyeongsang National University Hospital, Jinju, South Korea; 3 Institute of Health Science, Gyeongsang National University, Jinju, South Korea

**Keywords:** Insomnia, Mortality, Serum sodium, Hyponatremia

## Abstract

**Background:**

The association between lower serum sodium levels and the clinical outcomes of insomnia patients remains unclear. We explored whether lower serum sodium is associated with poor clinical outcomes in patients with insomnia.

**Methods:**

We retrospectively enrolled patients with a diagnosis of insomnia from January 2011 to December 2012. We divided participants into three groups according to initial serum sodium level: tertile 1 (< 138 mmol/L), tertile 2 (138.0–140.9 mmol/L), and tertile 3 (≥ 141.0 mmol/L). To calculate the relative risk of death, hazard ratios (HRs) and 95% confidence intervals (CIs) were obtained using Cox proportional hazard models.

**Results:**

A total of 412 patients with insomnia were included, of whom 13.6% (*n* = 56) had hyponatremia. Patients with lower serum sodium concentrations were older and had lower hemoglobin, calcium, phosphorus, and albumin levels. At the median follow-up of 49.4 months, 44 patients had died and 62 experienced acute kidney injury (AKI). Kaplan-Meier analysis showed significantly higher mortality in patients in the lowest tertile for serum sodium. The lowest tertile of the serum sodium level and the AKI were associated with all-cause mortality. However, the lowest tertile of the serum sodium level was not significantly associated with AKI.

**Conclusions:**

The lowest tertile of the serum sodium level was associated with a higher mortality rate in insomnia patients. Our results suggest that the serum sodium level could serve as a prognostic factor in insomniacs; patients with lower sodium levels require particular care.

## Background

Insomnia is a disorder characterized by at least one “nocturnal sleep symptom” and a daytime or “waking symptom” attributable to poor sleep [1]. The prevalence of insomnia in the general population is approximately 10–20%; about 50% of cases are chronic [2]. Insomnia is not only associated with a poor quality of life, but also with the risk of cognitive dysfunction [3], hypertension (HT) [4], metabolic diseases [5], and coronary artery disease (CAD) [6]. Although the pathophysiology of insomnia is complex, neurohormonal and sociocultural factors, as well as medical illnesses, are all associated with the condition. It is important to identify predisposing factors for insomnia.

Serum sodium level is important for neuronal function and osmoregulation between cells and the extracellular fluid [7]. Sodium is the main contributor to plasma osmolarity; certain disorders are typically characterized by hyponatremia and hypernatremia. To maintain optimal sodium concentrations, osmoreceptors in the hypothalamus and the kidneys tightly control water homeostasis [8]. Recent studies have shown that mild hyponatremia is associated with attention deficit, gait disturbance, and falls in patients admitted to the emergency room [9, 10]. Additionally, even mild hyponatremia is believed to be associated with risk of fracture [11] and mortality in adults living in the community [12–14].

Hospitalized hyponatremia patients usually exhibit several symptoms and signs that can be managed. Insomnia is one of the most intractable symptoms, usually associated with poor clinical outcomes. During the control of insomnia, we discovered that it was commonly accompanied by hyponatremia. We thus conducted the present study to identify factors that cause hyponatremia, and the clinical outcomes. We hypothesized that lower serum sodium levels would be associated with important clinical outcomes such as overall mortality and acute kidney injury (AKI) in insomnia patients.

## Methods

### Study population

This study retrospectively enrolled 774 insomniac adults (aged ≥18 years) admitted to Gyeongsang National University Hospital between January 2011 and December 2012. The inclusion criteria were: the presence of ICD-10-CM code G470 on the discharge form, and a prescription for insomnia (benzodiazepines, benzodiazepine receptor agonists, or melatonin). Data on demographic and clinical characteristics, laboratory findings, and comorbidities were obtained from the medical records at the time of admission. Patients with no available data on serum sodium level, and those who underwent renal replacement treatment, were being treated for cancer or had a history of cancer, or were lost to follow-up within 3 months were excluded. The follow-up time was the interval between the first and last hospital visits (collected from medical records), between admission and death.

### Definitions and clinical outcome measurements

Hyponatremia and hypernatremia were defined as a serum sodium level below 135 and above 145 mmol/L, respectively. Serum sodium level was corrected based on the serum glucose level in patients with hyperglycemia; the corrected sodium level was calculated as measured sodium + [(serum glucose - 100) × 0.016] [15]. The estimated glomerular filtration rate (eGFR) was calculated using the Modification of Diet in Renal Disease (MDRD) study formula [1.86 × (plasma creatinine) – 1.154 × (age) – 0.203)] × (0.74 if female) × (1.210 if black). Creatinine was measured using Jaffe one and serum sodium level was measured using an indirect ion-specific electrode. Chronic kidney disease (CKD) was defined as an eGFR < 60 mL/min/1.73 m^2^. AKI was defined as an increase in serum creatinine level ≥ 0.3 mg/dL within 48 h, or an increase in serum creatinine to ≥1.5 times the baseline value, either documented or presumed to have occurred within the previous 7 days.

Comorbidities were defined based on the International Classification of Diseases 10th Revision (ICD-10). Symptom severity was scored using the Charlson Comorbidity Index (CCI) [16]. Patients were divided into three groups: mild (CCI score = 1–2); moderate (CCI score = 3–4); and severe (CCI score ≥ 5) [17]. AKI was defined as an increase in serum creatinine level by ≥0.3 mg/dL within 48 h, or an increase in serum creatinine to ≥1.5-fold the baseline value that was either documented or presumed to have occurred within the previous 7 days [18]. We extracted mortality data from medical records, or from Statistics Korea if patients did not die while in our hospital [19]. To evaluate differences in demographic, laboratory and clinical outcomes data among insomnia patients, we divided them into tertile groups according to serum sodium level (tertile 1: < 138.0 mg/dL, tertile 2 [reference]: 138.0–140.9 mg/dL, tertile 3: ≥ 141.0 mg/dL). We also compared patients divided into hyponatremia and non-hyponatremia groups. The primary outcome was all-cause mortality and the secondary outcome was AKI incidence according to the tertiles of the serum sodium level. The study protocol was approved by the Institutional Review Board of Gyeongsang National University Hospital (IRB No. 2018–11–013-001).

### Statistical analysis

Data are presented as the mean ± standard deviation or frequency (count and percentage). Differences among the three tertile groups were determined using the chi-square test for categorical variables and analysis of variance (*t*-test) for continuous variables. To assess the association between serum sodium level and clinical factors, univariate and multivariate linear regression analyses were performed. To explore the association between serum sodium level and all-cause mortality, Kaplan-Meier curves were plotted for the three serum sodium groups. We also fit a restricted cubic spline function. Survival differences were compared using the log rank test. To calculate the relative risk of death, hazard ratios (HRs) and 95% confidence intervals (CIs) were derived based on Cox proportional hazards models. Factors showing a significant association (*P* <  0.10) after univariate analysis, or were of clinical concern, were included in Cox proportional hazards models. Variables were selected using a backward conditional method. Statistical analyses were performed using SPSS for Windows (ver. 21.0; SPSS Inc., Chicago, IL, USA) and R software (ver. 3.2.3; R Development Core Team, Vienna, Austria). Statistical significance was defined as *P* <  0.05.

## Results

### Baseline characteristics according to serum sodium level

A total of 412 patients were included in the final analysis: 362 patients were excluded for various reasons. A total of 148 patients (35.9%) were newly diagnosed with insomnia. The distribution of serum sodium levels is shown in Supplementary Fig. 1. The proportion of patients with hypernatremia was much lower than that with hyponatremia. The serum sodium levels were typically within the normal range. The mean age was 61.5 years and 56.1% (*n* = 231) of the patients were male. The mean follow-up duration was 49.4 months. The mean serum sodium level was 138.9 mmol/L. Patients with the lowest serum sodium levels (tertile 1) had significantly lower hemoglobin, calcium, phosphorus, total protein, albumin, and uric acid levels than the other two groups. Body mass index (BMI), systolic blood pressure (SBP), and heart rate (HR) did not differ significantly among the serum sodium tertiles. In tertile 1, the CCI score (≥ 5) was significantly higher than that of the other groups. However, the number of patients taking thiazide medications did not differ among the tertiles (Table 1). The percentage of patients with hyponatremia, defined as < serum sodium 135 mmol/L, was 13.6% (*n* = 56). Baseline characteristics of the insomnia patients according to hyponatremia status are shown in Supplementary Table. The hyponatremia group had significantly lower serum hemoglobin, calcium, albumin, and cholesterol levels, as well as a shorter follow-up duration and a higher proportion of CCI scores ≥5, than the non-hyponatremia group. Fifteen insomniacs (3.6%) had chronic hyponatremia; their mortality rate was significantly higher than that of the other patients (46.7% vs. 9.3%, *P* <  0.001).

**Table 1:** Baseline characteristics of insomnia patients by tertiles of serum sodium levels

Variables	Total (***N*** = 412)	< 138.0 mg/dL (***N*** = 147)	138.0–140.9 mg/dL (***N*** = 136)	≥141.0 mg/dL (***N*** = 129)	***P***
Age (yr)	61.5 ± 14.8	63.4 ± 14.2	60.4 ± 16.1	60.4 ± 13.8	0.150
Men (%)	231 (56.1)	93 (63.3)	77 (56.6)	61 (47.3)	0.028
Body mass index (kg/m^2^)	23.5 ± 2.2	23.3 ± 2.0	23.6 ± 2.3	23.6 ± 2.2	0.431
Systolic blood pressure (mmHg)	124.2 ± 12.1	123.8 ± 11.9	124.7 ± 12.1	124.1 ± 12.5	0.826
Diastolic blood pressure (mmHg)	79.8 ± 7.8	79.9. ± 7.3	80.0 ± 8.0	79.5 ± 8.1	0.906
Serum sodium, (mmol/L)	138.9 ± 3.7	135.0 ± 2.9	139.6 ± 0.9	142.5 ± 1.6	< 0.001
Serum potassium (mmol/L)	4.1 ± 0.5	4.2 ± 0.5	4.2 ± 0.5	4.1 ± 0.5	0.570
Hemoglobin (g/dL)	12.6 ± 2.0	12.2 ± 1.9	12.8 ± 2.0	12.7 ± 2.0	0.012
Calcium (mg/dL)	8.9 ± 0.7	8.7 ± 0.6	9.0 ± 0.6	9.0 ± 0.6	< 0.001
Phosphorus (mg/dL)	3.5 ± 0.8	3.4 ± 0.9	3.6 ± 0.7	3.7 ± 0.7	0.025
Glucose (mg/dL)	129.9 ± 50.2	137.7 ± 59.6	130.9 ± 46.4	120.1 ± 40.1	0.015
Total Protein (g/dL)	6.5 ± 0.8	6.3 ± 0.8	6.6 ± 0.8	6.5 ± 0.7	0.043
Albumin (g/dL)	3.8 ± 0.7	3.5 ± 0.7	4.0 ± 0.7	4.0 ± 0.6	< 0.001
Cholesterol (mg/dL)	165.9 ± 45.2	159.6 ± 52.6	170.9 ± 41.7	171.5 ± 37.4	0.007
Uric acid (mg/dL)	4.6 ± 1.7	4.0 ± 1.9	4.8 ± 1.5	5.0 ± 1.6	< 0.001
eGFR (mL/min/1.73m^2^)	88.3 ± 24.8	89.0 ± 28.1	88.4 ± 22.4	87.4 ± 22.4	0.787
Follow up duration (month)	49.4 ± 29.0	42.5 ± 29.2	53.8 ± 27.7	52.9 ± 28.9	0.001
Charlson Comorbidity Index (CCI) Score					< 0.001
CCI score 0–2 (%)	161 (39.1)	36 (24.5)	61 (44.9)	64 (49.6)	
CCI score 3–4 (%)	145 (35.2)	56 (38.1)	47 (34.6)	42 (32.6)	
CCI score ≥ 5 (%)	106 (25.7)	55 (37.4)	28 (20.6)	23 (17.8)	
Use of thiazide (%)	32 (7.8)	12 (8.2)	11 (8.1)	9 (7.0)	0.920

*eGFR* estimated glomerular filtration rate

### Clinical parameters affecting the serum sodium level

We measured parameters affecting the serum sodium level in the insomnia patients. On univariate analysis, the male sex; and the hemoglobin, calcium, uric acid, albumin, and cholesterol levels were positively correlated with the serum sodium level; the CCI score and use of thiazides were negatively correlated. However, age, BMI, SBP, DBP, and HR were not significantly associated with the serum sodium level. On backward multivariate linear modeling, the uric acid and albumin levels were significantly associated with the serum sodium level (Table 2).

**Table 2:** Relationship between serum sodium and clinical parameters in insomnia patients

	Univariable		Multivariable	
	β	***P***	β	***P***
Age (yr)	−0.02	0.072	0.02	0.055
Sex (ref. male)	0.92	0.013	0.85	0.057
Hemoglobin (mg/dL)	0.23	0.011	0.03	0.824
Calcium (mg/dL)	1.16	< 0.001	−0.52	0.272
Phosphorus (mg/dL)	0.45	0.095	−0.18	0.542
Glucose (g/dL)	−0.01	0.086	−0.01	0.462
Uric acid (mg/dL)	0.35	0.003	0.30	0.031
Albumin (g/dL)	1.65	< 0.001	1.71	< 0.001
Cholesterol (mg/dL)	0.02	< 0.001	0.01	0.619
Charlson Comorbidity Index Score	−0.36	< 0.001	−0.23	0.107
Use of thiazide	−0.03	< 0.001	−0.24	0.749

*β* regression coefficient with serum sodium level, *eGFR* estimated glomerular filtration rate

Adjusted R-squared: 0.1019, AIC = 1810.452, BIC = 1837.088

Adjusted R-squared: 0.1149, AIC = 1820.215, BIC = 1843.045

### Prediction of all-cause mortality based on the serum sodium level

**Fig. 1:**
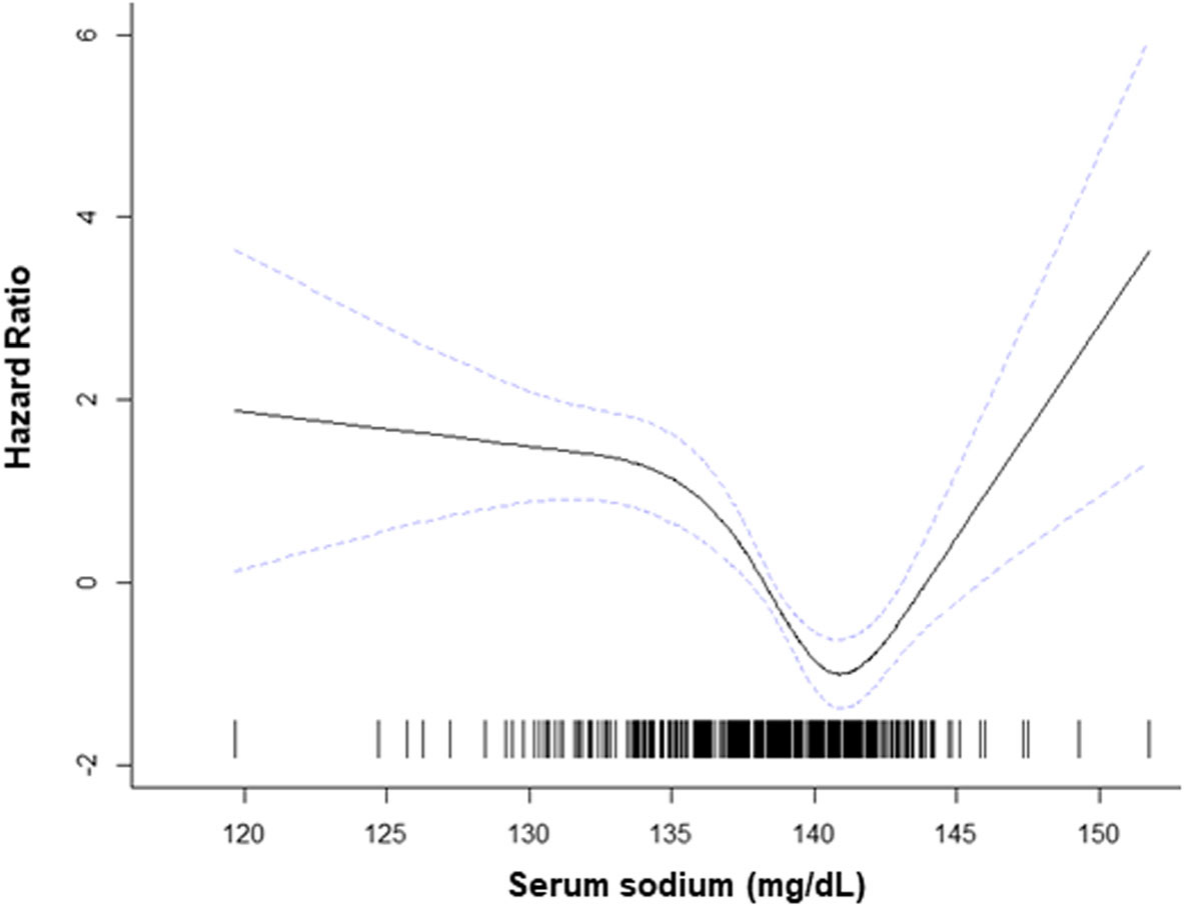
Association between serum sodium level and hazard ratios for all-cause mortality. The log hazard ratios for all-cause mortality (solid line) and 95% confidence index (dashed lines) are presented. Knots were located at serum sodium values of 137.7 and 140.8 mmol/l, corresponding to the 35th, and 70th percentiles

**Fig. 2:**
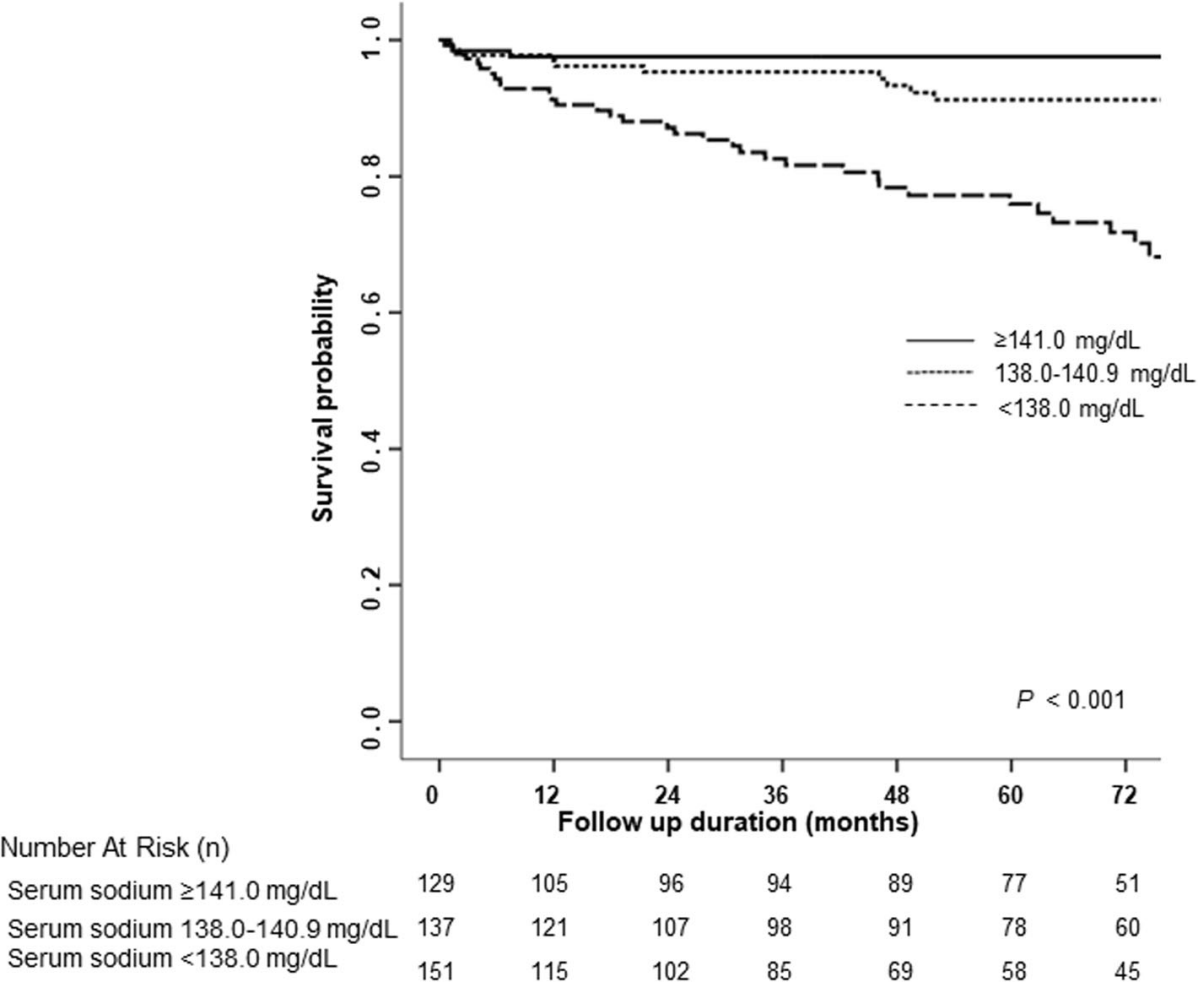
Kapan-Meier analysis of survival probabilities for tertile of serum sodium level The English in this document has been checked by at least two professional editors, both native speakers of English. For a certificate, please see: http://www.textcheck.com/certificate/iLexyH

We evaluated factors associated with all-cause mortality. During the median follow-up of 49.4 months, 44 (10.7%) patients died. We also examined how the risk of death varies with the overall serum sodium level. Figure 1 illustrates the nonlinear mortality risk according to the serum sodium level after adjusting for clinical covariates such as age, sex, hemoglobin, albumin, eGFR, and CCI score. There was a U-shaped association between serum sodium level and adjusted log-hazards ratio (HR). The HR was lowest at a serum concentration of 140–143 mg/dL; outside of this range, the HR increased in both directions (Fig. 1). The association of serum sodium tertile with all-cause mortality was evaluated using Kaplan-Meier analysis (Fig. 2). The results showed a significant difference in all-cause mortality among tertile groups. The lowest tertile of serum sodium (< 138.0 mg/dL) had a significantly higher mortality rate compared than the other two tertile groups. To explore the effect of serum sodium level on all-cause mortality, we performed Cox regression analyses. In multivariate analysis, being in the lowest serum sodium group (tertile 1; HR, 2.99 [95% CI: 1.40–6.39]) was an independent predictor of all-cause mortality in the insomnia patients, even after adjusting for all covariates (Table 3). In addition, the AKI (HR, 3.70 [95% CI: 1.99–6.90]) was significantly associated with all-cause mortality (Table 3).

**Table 3:** Hazard ratios for all-cause mortality risk factors in insomnia patients

	All-cause mortality	
	HR (95% CI)	***P***
Tertiles of serum sodium (ref. serum sodium 138.0–140.9 mg/dL)		< 0.001
Serum sodium < 138.0 mg/dL	2.99 (1.40–6.39)	
Serum sodium ≥141.0 mg/dL	0.36 (0.42–1.03)	
Acute kidney injury (ref. No)	3.70 (1.99–6.90)	< 0.001

*HR* hazard ratio, *CI* confidence interval

Adjusted for age, Hemoglobin, albumin, tertiles of serum sodium, Charlson comorbidity index score, acute kidney injury

AIC = 468.49, BIC = 473.84, AUC(c-index) = 0.742

### Prediction of acute kidney injury

Acute kidney injury occurred in 15.0% (*n* = 62) of patients. Table 4 summarizes the results of multivariate logistic regression analyses. The albumin level (HR 0.48 [95% CI: 0.31–0.76]), eGFR (HR, 0.98 [95% CI: 0.96–0.99], and CCI score (HR, 1.26 [95% CI: 1.06–1.51]) were significantly associated with AKI. However, the lowest serum sodium level tertile was not significantly associated with AKI.

**Table 4:** Odds ratio for acute kidney injury risk factors in insomnia patients

	AKI	
	OR (95% CI)	***P***
Albumin (g/dL)	0.48 (0.31–0.76)	0.002
Estimated glomerular filtration rate (mL/min/1.73m^2^)	0.98 (0.96–0.99)	< 0.001
Charlson comorbidity index score	1.26 (1.06–1.50)	0.008

*OR* odds ratio, *CI* confidence interval

Adjusted for age, hemoglobin, albumin, eGFR, tertiles of serum sodium, Charlson comorbidity index score

AIC = 323.13, BIC = 343.24, AUC (C-index) = 0.715

## Discussion

Our study showed a U-shaped relationship between overall serum sodium level and mortality and the lowest tertile of serum sodium was significantly associated with increased all-cause mortality even after adjusting for covariates. To the best of our knowledge, our results are the first to demonstrate an independent association between serum sodium level and all-cause mortality in insomnia patients.

The association between lower serum sodium level and insomnia has not been studied previously. We hypothesized that insomnia may be associated with a lower serum sodium level for the following reasons: first, the comorbidities of patients with insomnia may themselves be associated with lower serum sodium levels. In our study, 61.4% (*n* = 258) of patients had comorbidities, the most common of which was HT, followed by cardiovascular disease (CVD), chronic respiratory disease (CRD), and DM. These comorbidities are known to be associated with decreased serum sodium levels and commonly cause hyponatremia. In other words, lower serum sodium levels may not be due to insomnia itself, but rather to comorbidities. Second, activated sympathetic nerve activity due to insomnia [20, 21], leading to increased renin release [22, 23] and tubular fluid reabsorption [24], may be associated with low serum sodium.

Previous studies indicated that hyponatremia is an independent predictor of increased mortality in the general population [21], as well as in patients with a variety of diseases such as acute ST-elevation myocardial infarction [25], heart failure [26], and liver disease [27]. It has not yet been determined whether hyponatremia is simply an indicator of disease severity, or itself affects the disease. Chawla et al. suggested that serum sodium is seldom the cause of death but rather a marker of the severity of underlying disease [28]. Another study suggested that hyponatremia is an independent predictor of mortality even after adjusting for age, gender, and several comorbidities in the general outpatient population [29].

In our study of patients with insomnia, the lowest serum sodium tertile had the highest risk of all-cause mortality. The exact mechanism underlying increased mortality in these patients remains unclear. However, it is possible that activation of the autonomic nervous system in insomnia patients could be associated with both lower serum sodium levels and increased mortality risk. Hyperarousal is also considered a key pathophysiological mechanism in insomnia [1], increasing the whole-body metabolic rate during sleep, high-frequency electroencephalographic activity during non-rapid eye movement sleep, and cortisol and adrenocorticotropic hormone levels during the early sleep period, and decreasing parasympathetic tone and HR variability [30, 31]. These hyperarousal states may be associated with increased cardiovascular activity, and insomnia is known to be associated with both CVD risk and mortality [32]. We found that the HR was related to sympathetic nerve activity, but there was no significant association of the HR with the serum sodium level or mortality. We enrolled only hospitalized patients; in general, it would be difficult to identify sympathetic nerve activity using the HR alone.

Another hypothesis is that hyponatremia may be associated with various medical conditions including bone fractures, falls [9, 10], cardiovascular events [33], and cognitive dysfunction [3, 34], eventually leading to a high mortality rate [29]. Our study showed that the lowest tertile of serum sodium had a higher proportion of comorbidities, although not statistically significantly. Also, certain demographic, hematologic, and biochemical parameters, such as older age and lower serum hemoglobin, calcium, phosphorus, protein, albumin, and uric acid levels, were commonly seen in our insomnia patients in the lowest tertile of serum sodium. These variables also had a direct or indirect impact on mortality.

Notably, all-cause mortality was significantly associated with CRD in insomnia patients. Previous studies found that poor sleep quality was common among patients with chronic obstructive pulmonary disease (COPD) [35, 36], and that disturbed sleep was associated with mortality and adverse COPD outcomes [37]. Severe hypoxemia was observed during sleep in COPD patients [38]; this condition not only causes insomnia, but may also be associated with poor clinical outcomes. Consistent with previous studies, we found that inpatients with insomnia and underlying CRD had a high mortality rate. Thus, if a CRD patient complains of insomnia, exacerbation of respiratory disease should be suspected and treated if necessary.

Previous studies have shown that hyponatremia is associated with the development of AKI in hospitalized patients [39]. Other reports have suggested that hyponatremia is a significant prognostic factor for renal replacement therapy in CKD patients treated with diuretics, eventually leading to AKI [40]. Furthermore, one report showed that serum sodium itself would not have a significant effect on kidney function [41]. However, no study has explored the relationship between AKI incidence and lower serum levels in insomnia patients. We hypothesized that lower serum is associated with AKI in insomnia patients. However, we could not demonstrate a significant relationship between these two factors. Rather, we found that the serum albumin level, the estimated glomerular filtration rate, and the CCI score were associated with AKI in insomnia patients. The lack of a relationship between low serum sodium and AKI may be related to the cause of the AKI, such as volume depletion, toxic agents, ischemic conditions, or obstruction, as well as the severity of the AKI.

Interestingly, we found a higher hazard ratio for mortality in the lowest serum sodium level tertile (i.e., even in patients within the normal sodium range). Previous studies reported that such patients experienced poor clinical outcomes, including hepatic encephalopathy, mortality, and cognitive degeneration [3, 42]. This may indicate that a low but “normal” serum sodium level reflects an underlying condition with implications for mortality. We cannot definitively conclude that increasing the sodium levels of such patients would be effective; there are many other factors that need to be identified and controlled prior to interventional trials. Similarly, it may be necessary to control insomnia prior to sodium supplementation.

There were several limitations to our study. First, since it used a single-center retrospective design and relied on data from medical records, we could not tightly control certain factors that may affect the serum sodium level such as volume status, drugs (excluding thiazide), and hormone levels, and our results may thus not be generalizable. Second, we obtained serum sodium levels at baseline only; we could not obtain them at follow-up. Therefore, we could not monitor changes in the serum sodium level. Third, we enrolled insomnia patients based only on the ICD code and did not use other tools such as polysomnography or sleep habit questionnaires. However, we believe that these limitations were ameliorated by the large number of patients enrolled and the use of robust statistical methods. Relatively similar laboratory tests were applied and patients were followed-up at the same facility, since this was a single-center study.

## Conclusions

Our study showed a relationship between lower serum sodium levels and mortality in insomnia patients. The lowest tertile of serum sodium level was associated with mortality in these patients. Further studies are required to explore how insomnia, a low serum sodium level, and poor clinical outcomes are associated. Physicians should consider serum sodium as a prognostic factor in patients with insomnia.

## Supplementary information

**Additional file 1: Supplementary Table.** Baseline characteristics of insomnia patients by hyponatremia

**Additional file 2.**

## Data Availability

The datasets used and/or analyzed during this study are available from the corresponding author on reasonable request.

## Abbreviations

AKI: Acute kidney injury
BMI: Body mass index
CAD: Coronary artery disease
CCI: Charlson comorbidity index
CKD: Chronic kidney disease
COPD: Chronic obstructive pulmonary disease
CRD: Chronic respiratory disease
CVD: Cardiovascular disease
DBP: Diastolic blood pressure
DM: Diabetes mellitus
eGFR: Estimated glomerular filtration rate
HR: Heart rate
HT: Hypertension
SBP: Systolic blood pressure
