# Supplementary material for: Lower serum sodium levels predict poor clinical outcomes in patients with insomnia
Source: BMC Nephrol. 2020 Sep 5;21:386. doi: 10.1186/s12882-020-02051-w (PMC7487902; doi:10.1186/s12882-020-02051-w)
Supplement: Supplementary file 1 — Additional file 1: Supplementary Table. Baseline characteristics of insomnia patients by hyponatremia [file 12882_2020_2051_MOESM1_ESM.docx]

**Supplementary Table. Baseline characteristics of insomnia patients by hyponatremia**

| **Variables** | **Total**  **(N = 412)** | **Non-hyponatremia**  (N=356) | **Hyponatremia**  (N=56) | ***P*** | |  |
| --- | --- | --- | --- | --- | --- | --- |
| Age (yr) | 61.5 ± 14.8 | 61.1 ± 15.0 | 64.0 ± 13.2 | 0.177 | |  |
| Men (%) | 231 (56.1) | 195 (54.8) | 36 (64.3) | 0.183 | |  |
| Body mass index (kg/m^2^) | 23.5 ± 2.2 | 23.5 ± 2.2 | 23.5 ± 2.0 | 0.847 | |  |
| Systolic blood pressure (mmHg) | 124.2 ± 12.1 | 124.1 ± 12.1 | 124.8 ± 12.1 | 0.731 | |  |
| Diastolic blood pressure (mmHg) | 79.8 ± 7.8 | 79.7 ± 7.8 | 80.2 ± 7.6 | 0.681 | |  |
| Serum sodium, (mmol/L) | 138.9 ± 3.7 | 139.9 ± 2.6 | 132.2 ± 2.8 | <0.001 | |  |
| Serum potassium (mmol/L) | 4.1 ± 0.5 | 4.1 ± 0.5 | 4.2 ± 0.6 | 0.313 | |  |
| Hemoglobin (g/dL) | 12.6 ± 2.0 | 12.7 ± 2.0 | 11.6 ± 1.9 | <0.001 | |  |
| Calcium (mg/dL) | 8.9 ± 0.7 | 8.9 ± 0.6 | 8.6 ± 0.7 | 0.001 | |  |
| Phosphorus (mg/dL) | 3.5 ± 0.8 | 3.6 ± 0.8 | 3.5 ± 0.9 | 0.551 | |  |
| Glucose (mg/dL) | 129.9 ± 50.2 | 129.2 ± 47.4 | 134.7 ± 65.4 | 0.442 | |  |
| Total Protein (g/dL) | 6.5 ± 0.8 | 6.5 ± 0.7 | 6.3 ± 0.9 | 0.095 | |  |
| Albumin (g/dL) | 3.8 ± 0.7 | 3.9 ± 0.6 | 3.3 ± 0.6 | <0.001 | |  |
| Cholesterol (mg/dL) | 165.9 ± 45.2 | 168.7 ± 39.7 | 148.1 ± 68.0 | 0.002 | |  |
| Uric acid (mg/dL) | 4.6 ± 1.7 | 4.6 ± 1.6 | 4.6 ± 2.4 | 0.778 | |  |
| eGFR (mL/min/1.73m^2^) | 88.3 ± 24.8 | 89.1 ± 23.4 | 85.7 ± 30.1 | 0.337 | |  |
| Follow up duration (month) | 49.4 ± 29.0 | 51.3 ± 28.4 | 37.7 ± 30.3 | 0.001 | |  |
| Charlson Comorbidity Index (CCI) Score |  |  |  | <0.001 | |  |
| CCI score 0-2 (%) | 161 (39.1) | 150 (42.1) | 11 (19.6) |  | |  |
| CCI score 3-4 (%) | 145 (35.2) | 125 (35.1) | 20 (35.7) |  | |  |
| CCI score ≥5 (%) | 106 (25.7) | 81 (22.8) | 25 (44.6) |  | |  |
| Use of thiazide (%) | 32 (7.8) | 29 (8.1) | 3 (5.4) | | 0.341 | |

eGFR, estimated glomerular filtration rate
