# Supplementary figures and images for: Lower serum sodium levels predict poor clinical outcomes in patients with insomnia
Source: BMC Nephrol. 2020 Sep 5;21:386. doi: 10.1186/s12882-020-02051-w (PMC7487902; doi:10.1186/s12882-020-02051-w)

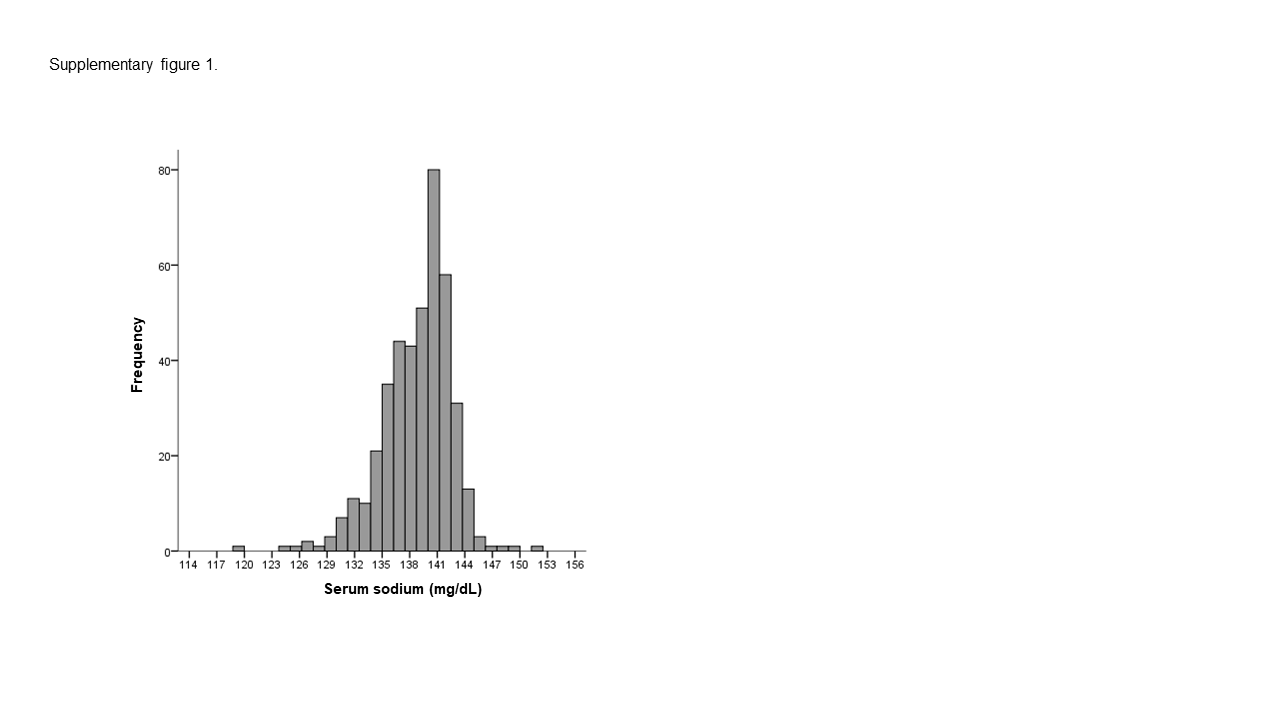

Supplement: Supplementary file 2 — Additional file 2. [file 12882_2020_2051_MOESM2_ESM.tif]
